# Mass Cytometry Defines Virus-Specific CD4^+^ T Cells in Influenza Vaccination

**DOI:** 10.4049/immunohorizons.1900097

**Published:** 2020-12-11

**Authors:** Priyanka B. Subrahmanyam, Tyson H. Holmes, Dongxia Lin, Laura F. Su, Gerlinde Obermoser, Jacques Banchereau, Virginia Pascual, Adolfo García-Sastre, Randy A. Albrecht, Karolina Palucka, Mark M. Davis, Holden T. Maecker

**Affiliations:** * Institute for Immunity, Transplantation, and Infection, Stanford University School of Medicine, Stanford, CA 94305; † Baylor Institute for Immunology Research, Baylor Research Institute, Dallas, TX 75246; ‡ The Jackson Laboratory for Genomic Medicine, Farmington, CT 06032; § Department of Microbiology, Icahn School of Medicine at Mount Sinai, New York, NY 10029; ¶ Division of Infectious Diseases, Department of Medicine, Icahn School of Medicine at Mount Sinai, New York, NY 10029; ǁ Global Health and Emerging Pathogens Institute, Icahn School of Medicine at Mount Sinai, New York, NY 10029

## Abstract

The antiviral response to influenza virus is complex and multifaceted, involving many immune cell subsets. There is an urgent need to understand the role of CD4^+^ T cells, which orchestrate an effective antiviral response, to improve vaccine design strategies. In this study, we analyzed PBMCs from human participants immunized with influenza vaccine, using high-dimensional single-cell proteomic immune profiling by mass cytometry. Data were analyzed using a novel clustering algorithm, denoised ragged pruning, to define possible influenza virus–specific clusters of CD4^+^ T cells. Denoised ragged pruning identified six clusters of cells. Among these, one cluster (Cluster 3) was found to increase in abundance following stimulation with influenza virus peptide ex vivo. A separate cluster (Cluster 4) was found to expand in abundance between days 0 and 7 postvaccination, indicating that it is vaccine responsive. We examined the expression profiles of all six clusters to characterize their lineage, functionality, and possible role in the response to influenza vaccine. Clusters 3 and 4 consisted of effector memory cells, with high CD154 expression. Cluster 3 expressed cytokines like IL-2, IFN-γ, and TNF-α, whereas Cluster 4 expressed IL-17. Interestingly, some participants had low abundance of Clusters 3 and 4, whereas others had higher abundance of one of these clusters compared with the other. Taken together, we present an approach for identifying novel influenza virus–reactive CD4^+^ T cell subsets, a method that could help advance understanding of the immune response to influenza, predict responsiveness to vaccines, and aid in better vaccine design.

## INTRODUCTION

Influenza virus infection is a serious health concern, especially in children, elderly patients, and those with respiratory disorders or other chronic medical conditions. The influenza vaccine is important to protect the general population from contracting the disease and is especially critical for high-risk groups who will need more frequent hospitalizations and suffer complications from infection and even mortality. Unfortunately, the current influenza vaccine must be developed, produced, and administered every year, based on the predicted reassortment of viral strains for that specific year. The effectiveness of the vaccine can vary drastically by viral strain from season to season and among different age and risk groups (1–3).

Conventionally, the immune response to influenza virus has been characterized by the B cell–mediated production of virus-specific neutralizing or agglutinating IgG Abs. Hence, the hemagglutination inhibition (HAI) assay is widely used to measure protective immunity to influenza virus. Most vaccine development and evaluation has thus focused on this arm of the immune system. However, in light of the limited effectiveness of the current influenza vaccine, there is a need to diversify vaccine design approaches, possibly by including strategies for T cell activation (4–7). To this end, there is great interest in studying the role of T cells, especially CD4^+^ T cells, in the generation and shaping of the immune response to influenza virus (8–12). CD4^+^ T cells play a multifaceted role in the antiviral response to influenza viruses, including the important aspect of B cell help provided by T follicular helper (Tfh) cells (13, 14). First, CD4^+^ T cells provide B cell help for the initiation of germinal centers and the generation of high-affinity Abs (15). Furthermore, they are also important in the generation and expansion of CD8^+^ memory T cell subsets, which can mount effective cytotoxic responses to virally infected cells (16, 17). Finally, a subset of cytolytic CD4^+^ T cells have been shown to be protective in influenza and could serve as an additional avenue to boost the immune response (6, 18). Because CD4^+^ T cells are a vastly heterogeneous population, it is imperative to identify specific subsets that are key players in immunity to influenza virus. The frequency of T cells specific for influenza virus Ags in blood is low, making it important to determine that the cells being studied are actually vaccine responsive, including those that arise from naive T cells, as well as pre-existing memory T cells generated during previous vaccination and/or exposure to influenza viruses (19). Such efforts can eventually guide vaccine design to create the next generation of more effective and broadly reactive influenza vaccines.

**FIGURE 1.**
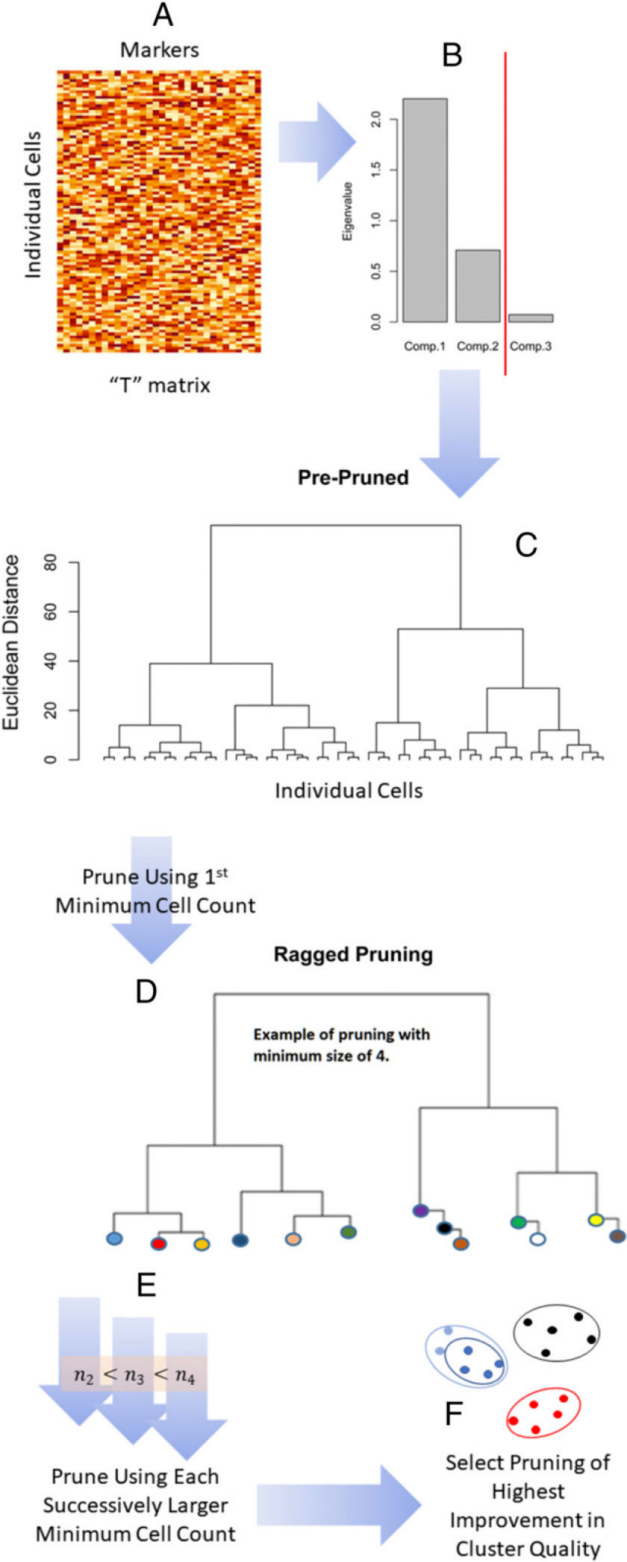
Schematic representation of DRP algorithm. (**A**) The original data matrix has individual cells as rows and cell surface markers as columns. (**B**) A principal components analysis is performed on this original data matrix, and the data matrix is denoised by hard eigenvalue thresholding (red line). (**C**) An initial tree is created based on an agglomerative hierarchical clustering of the denoised data. (**D**) This tree is then pruned back to create a set of clusters (circles of different colors) that equal or exceed the first minimum cell count (e.g., illustrated in this study with minimum of four). (**E**) This pruning process is repeated for each successively larger minimum cell count. (**F**) The optimal cluster solution is from that minimum cell count of greatest improvement in cluster quality relative to next smallest minimum cell count. Together, (D) and (F) illustrate the formation of nested clusters (see Materials and Methods). Actual trees will be many times larger than depicted in this study because total cell counts will equal or exceed *O* [10^3^]. For this reason, DRP is computer-memory demanding. The figure was generated using R package dendextend (64), R package MASS (65), base R (43), and Microsoft PowerPoint and Word and Windows Paint (Microsoft Corporation, Redmond, WA). See Statistical Methods and also see Section S1.2.2 in Bruggner et al. (24).

In this study, we performed a detailed phenotypic and functional characterization of PBMCs from donors before vaccination (day 0) and after vaccination (day 7) with trivalent inactivated influenza vaccine (TIV). Using mass cytometry by time of flight (CyTOF), we were able to perform high-dimensional single-cell immune profiling, with the goal of identifying influenza virus–specific immune cell subsets. In this study, we used a novel method called denoised ragged pruning (DRP, Fig. 1) for the specific purpose of determining influenza virus–specific CD4^+^ T cell phenotypes.

Several algorithms are in use for clustering of data acquired via mass cytometry [Table II in (20)]. DRP is specially designed to be applicable in small datasets (i.e., low total cell counts) in two important respects. First, DRP begins with a denoising step. A prominent area of active research in CyTOF data analysis is noise reduction, in which sources of noise are many and diverse (20). Denoising can be especially useful in small datasets (21). Denoising removes nonstructural variation in data without “selecting out” any markers from the analysis. Clustering that includes marker selection [e.g., as in (22)], although quite useful in general, is not always applicable in mass cytometry studies because too few rather than too many markers can be acquired, although in theory, “high levels of multiplexing (>40 proteins in parallel) are possible” (23). For example, ideally, the panel of markers in the current study might have included more markers than just ICOS for definitively identifying circulating Tfh cells. However, even a large CyTOF staining panel cannot accommodate all phenotyping and functional markers needed for dissecting the broad immune response reflected in peripheral blood. Second, as we demonstrate below, the DRP pruning algorithm permits isolation of rare phenotypes. This property might distinguish DRP from the related method of Citrus (24) because DRP includes an initial denoising step, a different method for ragged pruning (Fig. 1D), and optimal pruning. Differential analysis of cydar is an interesting alternative approach that may outperform Citrus for detecting phenotypes (25), deterministic spanning-tree progression analysis of density-normalized events (SPADE) employs outlier removal and systematic down weighting to facilitate detection of rare phenotypes (26), and X-shift’s weighted *k*-nearest-neighbor density estimation algorithm has been demonstrated to recover additional phenotypes not identified via manual gating as well as transitional phenotypes (27); however, neither cydar, SPADE, nor X-shift includes an initial denoising step to facilitate resolution of phenotypic structure, a step that may be crucial in the analysis of data consisting of low total cell counts. Indeed, DRP is designed for datasets containing ≲ 10,000 total cells.

**FIGURE 2.**
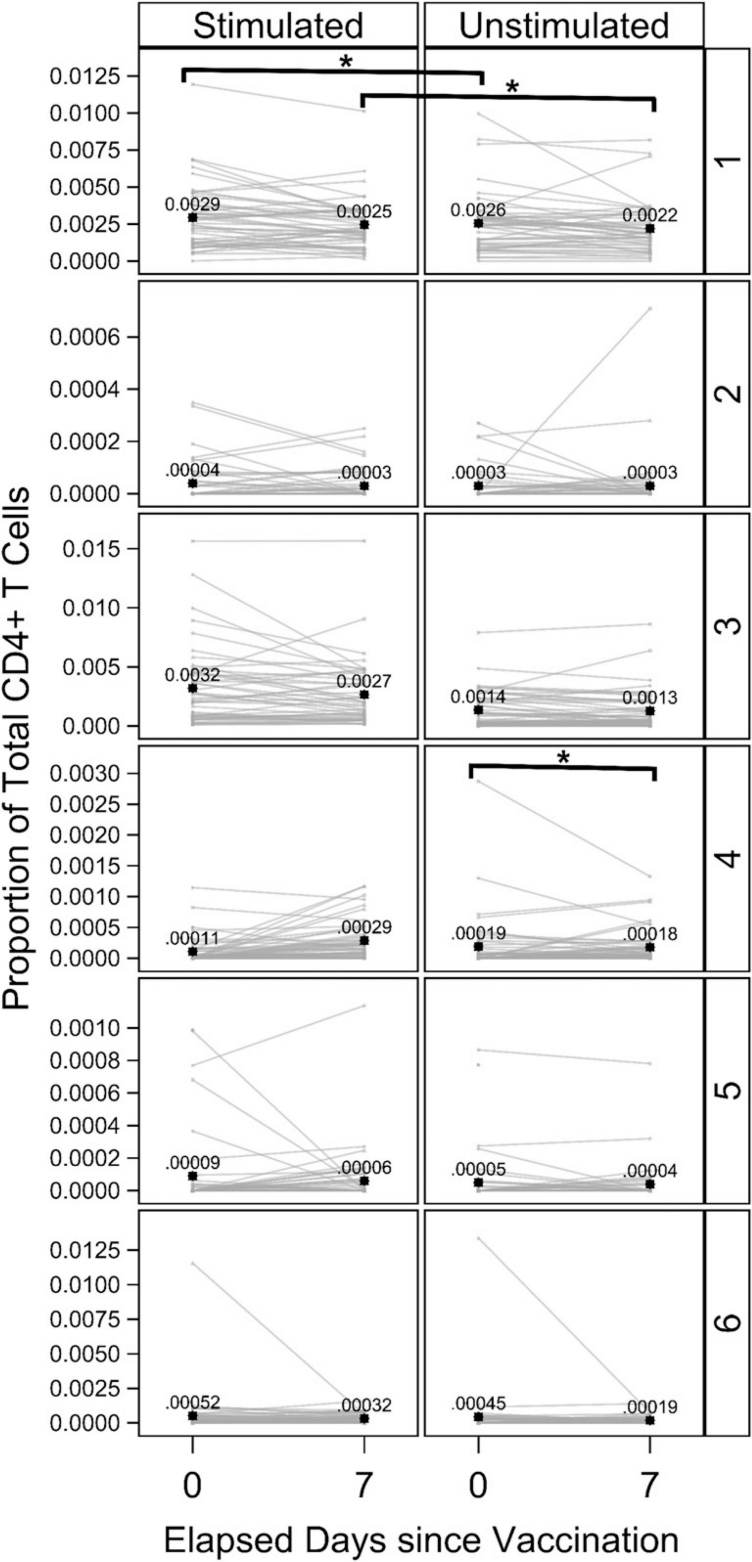
Estimated proportions of all six clusters identified by DRP. The estimated proportions of each cluster identified by DRP at day 0 and day 7 are represented graphically. Left panels show the influenza peptide–stimulated condition, and right panels show the unstimulated condition. Each line represents a single study participant. *p < 0.05. Black star symbols are estimated mean proportions for that cluster, stimulation condition, and day with the numeric values reported to four to five decimal places. The estimates of mean proportions differ from Table III because they are without correction for regression covariates. The figure was prepared in SAS ODS Graphics (SAS Institute).

The “denoised” component of the DRP algorithm (Fig. 1B) isolates and removes noise from the signal intensity dataset prior to clustering. Additionally, cell subsets of interest can vary considerably in relative abundances (e.g., Fig. 2, vertical axis). DRP’s “ragged-pruning” component (Fig. 1, Step D) permits estimation of all distinct cell phenotypes, whether represented by small or very large quantities of individual cells in the dataset. Taken together, these two targeted properties make DRP invaluable in the study of cell subsets that may be rare and of subtle but distinct variations. Using DRP, we were able to identify two relevant CD4^+^ T cell clusters from the CyTOF data, one of which appears to be a pre-existing influenza virus–specific cluster, and the other was an influenza vaccine-responsive cluster. We further characterized expression profiles of these clusters to understand their possible functional role in the immune response to influenza virus.

## MATERIALS AND METHODS

### Sample selection

Blood samples were collected from 46 healthy donors prior to (day 0) and 7d after (day7) influenza vaccination. All study participants were healthy donors, with samples collected under Institutional Review Board–approved influenza vaccine studies at Stanford University (SU; two separate studies) and Baylor Institute for Immunology Research (BIIR; one study). Age range was 12–80 y (median=42y); detailed age/gender information is shown in Table I. All participants received the seasonal TIV during the period of 2009–2012.

### PBMC collection and storage

Heparinized blood was subjected to Ficoll-Hypaque gradient separation, and PBMC were cryopreserved using standard protocols. Samples collected at BIIR were shipped to SU on dry ice, but all samples were otherwise stored in liquid nitrogen until thawing for CyTOF analysis, as described below.

### CyTOF intracellular cytokine staining assay

Cells were stained and prepared for CyTOF analysis as previously described (28). Briefly, frozen PBMC samples from participants were thawed and resuspended in complete medium (RPMI 1640 supplemented with 10% FBS, penicillin, streptomycin, and L-glutamine) with benzonase. After washing, cells were counted, and 2 × 10^6^ cells (or maximum available) were placed in a 96-well U-bottom plate in complete medium (benzonase-free). Cells were rested overnight at 37°C and 5% CO_2_. Cells were then stimulated for 8h with 1μg/ml each of hemagglutinin (HA) PepMix Influenza A California H1N1 (139 peptides) and NP PepMix Influenza A H3N2 (122 peptides), both from JPT Peptide Technologies (Berlin, Germany). We chose this HA+NP peptide mix because although current inactivated influenza virus vaccines are partly purified and standardized for their HA content, there are some levels of NP present in them. The secretion inhibitor monensin from Biolegend (San Diego, CA), 2 μg/ml anti-CD40 from Miltenyi Biotec (Bergisch Gladbach, Germany), and 1 μg/ml anti-CD28/CD49d from BD Biosciences (San Jose, CA) were also added (the latter for costimulation, the former to prevent CD40L downmodulation). Anti-CD107a labeled with ^154^Sm (conjugated in-house) was also added during stimulation to allow labeling of transiently expressed CD107a. After 4 h, 5 μg/ml brefeldin A from Sigma-Aldrich (St. Louis, MO) was added, and the plate was incubated for another 4 h at 37°C. Surface markers were stained using a mixture of metal ion-conjugated Abs diluted in CyFACS buffer (metal-free PBS from Rockland Immunochemicals [Pottstown, PA] with 0.1% BSA, 2mM EDTA, and 0.05% sodium azide) (Table II). Preconjugated Abs from Fluidigm (South San Francisco, CA), as well as in-house conjugated Abs, were included in the panel (Table II). ^115^In Maleimide-DOTA from Macrocyclics (Plano, TX) was used for LIVE/DEAD staining as per the supplier’s recommendations. Cells were fixed in 2% paraformaldehyde diluted in metal-free PBS from Rockland Immunochemicals and permeabilized using permeabilization buffer from eBioscience (Thermo Fisher Scientific, Waltham, MA). These fixed and permeabilized cells were stained using an intracellular Ab mixture diluted in permeabilization buffer (Table II). Finally, cells were stained with ^191^Ir and ^193^Ir DNA intercalator from Fluidigm, as per the manufacturer’s directions. Samples were washed twice in CyFACS and 3 times in Milli-Q water before running. EQ Four Element Calibration beads from Fluidigm were added to the sample as directed. Data were acquired on a CyTOF Version 1 instrument from Fluidigm.

### CyTOF data analysis

Raw data were obtained from CyTOF as .fcs files. Using the calibration beads, these data were normalized for instrument performance using the Nolan Lab MATLAB-based normalizer, which is freely available on Github (https://github.com/nolanlab/bead-normalization). Thereafter, the normalized .fcs files were loaded into FlowJo (Version 9.9.4) from Tree Star (Ashland, OR). Sequential gating of CyTOF data was performed as described previously (28). Briefly, gating on events positive for both DNA markers ^191^Ir and ^193^Ir (DNA1 and DNA2) was used to identify intact cells. DNA marker along with event length were used to gate intact singlets. We then used ^115^In Maleimide-DOTA (a dead cell stain) to gate on live intact singlets. Based on CD14 and CD33 expression, these live intact singlets were further gated into lymphocytes (CD14^-^CD33^-^) and monocytes (CD14^+^CD33^+^). CD3 expression on lymphocytes was used to identify CD3^+^ T cells. T cells were then gated as CD4^+^ and CD8^+^ (28). The CD4^+^ T cell population from each sample was further analyzed for the expression of five cytokines: IFN-γ, IL-2, IL-17, TNF-α, and MIP1β (Supplemental Fig. 1). Boolean logic was used to identify cells that express any one or more of these five cytokines. Expression data on all panel markers on each of these cytokine^+^ cells were exported and tabulated for further statistical analysis as described below. Statistical analyses on these gated cytokine^+^ CD4^+^ T cells were limited to 32 markers relevant to T cells. The B cell marker CD19 and monocyte markers CD14 and CD33 were used for basic lineage gating but excluded from further statistical analysis. Also, two poor-performing markers, IL-10 and FOXP3, were excluded prior to the initiation of these statistical analyses. The T cell–relevant markers used for analysis were as follows: CXCR3, CCR6, CD57, CD69, CD4, CD8, CD3, MIP1β, CD85j, CD45RA, CD38, TNF-α, Granzyme, CD107a, GMCSF, CD154, IL-2, IFN-γ, HLA-DR, Ki67, ICOS, IL-17, CD127, CD27, CCR7, PD1, CXCR5, IL-21, Perforin, CD16, CD56, and CD25.

### Statistical methods

#### Cluster analysis.

Separately for each of the 32 markers, raw intensity data *y* were transformed as *x* = Arsinh[y5] and then centered and scaled as $t=(x-\bar{x})/s$ for sample mean $\bar{x}$ and corresponding sample SD *s*. Altogether, these transformed marker data formed data matrix **T** (one column per marker, and one row for each cell) for analysis (Fig. 1A). This data matrix **T** was used to estimate cell clusters via the application of our newly developed DRP clustering algorithm. DRP consists of five basic steps as follows.1) Denoising (Fig. 1B) was performed to separate structural components (e.g., biological structure) from noise components (e.g., technical error) (21). Structure was defined as the principal components of data matrix **T** with eigenvalues exceeding the 90th percentile of the null eigenvalue distribution (29). (Each eigenvalue is the sample variance of its corresponding principal component (30). Large eigenvalues indicate the presence of structure in data. Null eigenvalues are small and arise from structureless noise.) 2) Agglomerative hierarchical clustering (31), a form of cluster analysis, was performed on the principal components of large eigenvalues (i.e., structural principal components) of data matrix **T** (Fig. 1C). 3) The resultant hierarchical clustering tree was “pruned” back in possibly ragged fashion (Fig. 1D). A tree is a graph (32) that, in this study, displays hierarchical relationships among clusters of cells. The “branch tips” of the tree are individual cells. Moving from the branch tips to the “trunk,” clusters of cells are sequentially merged into larger and larger clusters of more cells, ending at the trunk of the tree, in which all cells have merged into a single cluster. Pruning the branches of this tree creates pruned branch tips that, together, represent a set of (merged) clusters, with each cell occurring in one cluster only. 4) Pruning was repeated many times, each time with a progressively larger criterion for minimum quantity of cells per “pruned branched tip” (Fig. 1E). Minimum quantity of cells was varied from 10 cells to ~20% of input sample size in increments of 50 cells. A set of cells are assigned to a cluster when they first form a branch with a quantity of cells equaling or exceeding the minimum. For example, suppose the minimum size is four, and two cells branch from an existing cluster of four cells. Those two cells are assigned to their own cluster (e.g., two blue clustersin Fig. 1F) because2+4.>4. This facilitates discovery of rare phenotypes. 5) The final step identified that pruning yielded a cluster solution of the highest increase in average cluster quality (33) with an increase in that minimum quantity of cells (Fig. 1E). Optimal cluster solution is illustrated in a heat map of mean Arsinh-transformed expression by marker and cluster and in penalized supervised star plots of Arsinh-transformed expression for all markers together (34).

#### DRP reproducibility and computational speed.

DRP’s pruning for an optimal cluster solution is thorough and thereby computationally intensive. As such, we recommend running DRP on samples sizes that do not exceed ~10,000 cells. We achieved these cell counts through stratified random downsampling. Stratification was on each combination of study (two at SU and one at BIIR), batch, participant, visit, and stimulation condition. Stratified random sampling allowed us to achieve less inequality in cell counts input to DRP such that all strata would be weighted less unequally in the cluster solution (Supplemental Fig. 2A). Specifically, within each stratum, sampling was random without replacement (35) and with sample size per stratum being the smaller of 88 cells or a sampling percentage of 90%. This rule yielded a downsampling percentage of ~50% (i.e., approximately half of all available cells were analyzed via DRP) per run of DRP. The DRP algorithm was run on three separate stratified random 50% downsamplings to examine the reproducibility of results across different random downsamples. This resulted in three separate DRP cluster solutions (Supplemental Fig. 2B). In Supplemental Fig. 2B, for heat map labels at right, the first two digits are percentage of downsampling, third digit is downsampling identification number (1–3), and last digit(s) is(are) DRP cluster identification number within that downsampling (1, 2, 3, …). Quantities of DRP clusters varied among downsamplings, the highest at 18 clusters for the third downsampling. In dendrogram at left (Supplemental Fig. 2B), longer horizontal line segments indicate greater separation in marker expression among clusters at that level in the dendrogram. Vertical yellow line was approximately placed where this separation is greatest, and this line cuts the dendrogram in seven places, yielding six major combined clusters. Seventh rare isolated cluster marked by an “X” was excluded from further analyses (Supplemental Fig. 2B). Clusters are termed “combined” because of combining across closely related clusters from different downsamplings and combining closely related clusters within the same downsampling, with all combining accomplished by cutting the dendrogram at the vertical yellow line. Note that a given cell may occur in more than one downsampling and that admits the possibility that cells may occur in more than one of these six major combined clusters. With all three downsamplings combined and the one cluster marked with an X removed, the dataset contained 15,015 cells with 3,828 cells (~25%) appearing in more than one of the combined clusters because of the three random downsamplings. We dropped any cells that occurred with equal frequency in more than one cluster. For example, suppose a cell was randomly selected in two downsamplings but assigned to two different major combined clusters; that cell was dropped. The same rule applied to a cell assigned to three different major combined clusters. Together, these accounted for ~10% of the 15,015 cells. Any cell that only occurred in one major combined cluster (11,187cells, ~75%) was retained. Any cell that occurred in one major combined cluster twice and a different major combined cluster once was retained for analysis (2279 cells, ~15%) and assigned to that major combined cluster where it occurred twice (majorityvote). For each cell removed (numerator), we decreased the total CD4 count (denominator) by one.

#### Comparing cluster proportions.

From the estimated optimal cluster solution (Fig. 1F), numerators and denominators of cell counts were tabulated. Numerator *n* was the cell count for each combination of study, batch, participant, visit, stimulation condition, and cluster and zero for any such combination without cells. Denominator *d* was the gated total CD4^+^ T cell count. These yielded an estimated cluster proportion per combination of study, batch, participant, visit, and stimulation condition of *r* = *n/d*. For each cluster, sample sizes (of participants) were 48, 48, 47, and 47 for day 0 unstimulated, day 0 stimulated, day 7 unstimulated, and day 7 stimulated, so two data values were missing. A regression model was fit separately for each cluster. Observed proportion *r* was regressed on visit, stimulation condition, the interaction of visit and stimulation condition, study (to account for any otherwise unmeasured differences in the three sampled populations), elapsed days from start of study for batch (batch day), and the interaction of study and batch day. The interaction of visit and condition allowed differences in cluster proportions between conditions to vary with visit and the converse. The interaction of study and batch day allowed any trends in proportions over time across batches to vary among studies. Outcome *r* was modeled as a binomial proportion; however, unlike a standard binomial distribution in which the denominator is constant, the denominator *d* in this study varies among participants and stimulation conditions within participants. For this reason, we employed fractional logistic regression with a robust estimator of the variance (36, 37) and random coefficients (i.e., mixed-effects model) for participants. These random coefficients are additional predictor variables that account for participant-specific variation in mean *r* not explained by visit, stimulation condition, the interaction of visit and stimulation condition, study, batch day, and the interaction of study and batch day. From the fit of the regression model, the average participant’s difference in means of proportions *r* between 1) stimulation conditions within each visit and 2) visits within each stimulation condition were estimated. All *p* values were adjusted for multiple comparisons (38) across all comparisons (i.e., visit comparisons combined with stimulation comparisons for all clusters).

#### Cluster proportions association with clinical outcome.

Separately for each strain, HAI titer at day 28 was regressed on the stimulated proportion of each cluster *r* at day 0 (baseline), study, and baseline HAI titer. Baseline HAI titer was employed as a covariate rather than formulating the outcome as fold change (day 28 divided by baseline) to improve statistical power (39). Because a titer of *h* indicates that true titer falls somewhere in the half-closed interval [*h*, 2h), analysis employed regression methods for interval-censored outcome data (40, 41). In a separate, secondary analysis, for each strain, HAI titer at day 28 was regressed on the stimulated proportion of each cluster *r* at day 7, study, and baseline HAI titer. Missing CyTOF and HAI data were multiply imputed using fully conditional specification with predictive mean matching (42). Fifty complete datasets were generated 1) with missing values for HAI titer imputed using predictors of HAI strain, study, day 0, and day 7 proportions for all six clusters and 2) with missing values for day 7 proportions of all six clusters imputed using predictors of HAI strain, study, day 0 and day 28 HAI titers, and day 0 proportions for all six clusters.

#### Software.

Statistical analyses were performed in SAS (SAS Institute, Cary, NC), base R (43), and R packages cwhmisc (44), Weight-edCluster (45), heatmap3 (46), matrixcalc (47), plotrix (48), JPEN (49), sampling (50), VCA (51), rospca (52), and tsne (53). Additional software for generating graphics is listed in the figure legends.

## RESULTS

We used mass cytometry (CyTOF) to examine the phenotypic and functional markers of influenza virus–specific T cells prior to and 1 wk after vaccination with seasonal TIV (Tables I, II). PBMC were unstimulated or stimulated with HA+NP overlapping peptides prior to the CyTOF assay. CyTOF data were manually gated to obtain cytokine^+^ (IFN-γ, IL-2, IL-17, TNF-α, or MIP1β) CD4^+^ T cells (Supplemental Fig. 1). CyTOF panel marker expression data for each of these cells were exported, and DRP was used to identify influenza virus–specific CD4^+^ T cell clusters (Fig. 1).

### Influenza virus–specific CD4^+^ T cell clusters identified by DRP

Using the novel statistical method of DRP, we obtained an optimized cluster solution for the cytokine^+^ CD4^+^ T cells. A total of six clusters were identified by this method (Fig. 2). A tabulated list of estimated proportions of all six clusters at all time points and stimulation conditions is shown in Table III. Table III clearly quantifies the extreme rarity of these phenotypes (e.g., stimulation at 7d postvaccination generates approximately two cells of Cluster 2 per 10,000 CD4^+^ T cells). At each time point, we compared the abundance of each cluster in influenza virus peptide–stimulated samples to their unstimulated counterparts. Cluster 3 was significantly higher in influenza peptide–stimulated versus– unstimulated conditions (Table IV), suggesting that it is an influenza virus–specific cluster. Cluster 3 was significantly higher in the stimulated condition at both day 0 and day 7; thus, it appears to represent a memory T cell response to influenza viruses (which may or may not have been increased by vaccination). In contrast, Cluster 4 was significantly higher at day 7 after vaccination compared with day 0 in the influenza peptide–stimulated condition (Fig. 2, Table IV). This indicates that Cluster 4 is a vaccine-induced CD4^+^ T cell cluster that may play an important role in the immune response to the virus. All other clusters (Clusters 1, 2, 5, and 6) did not show significant differences between stimulated and unstimulated conditions or between the two time points (Table IV). They thus represented T cells that were cytokine producing, but not influenza virus specific, or that were so rare (e.g., Cluster 6) and/or variable among participants as to not reach statistically significant increases above their unstimulated background, given the number of cells sampled. A complete list of all comparisons across stimulation conditions and time points is shown in Table IV. Furthermore, a tabulated list of estimated proportions of all clusters at all time points and stimulation conditions is shown in Table III. Table III quantifies the extreme rarity of these phenotypes (e.g., stimulation at 7 d postvaccination generates approximately two cells of Cluster 2 per 10,000 CD4^+^ T cells).

### Immunophenotypic characterization of influenza virus–specific Clusters 3 and 4

**FIGURE 3.**
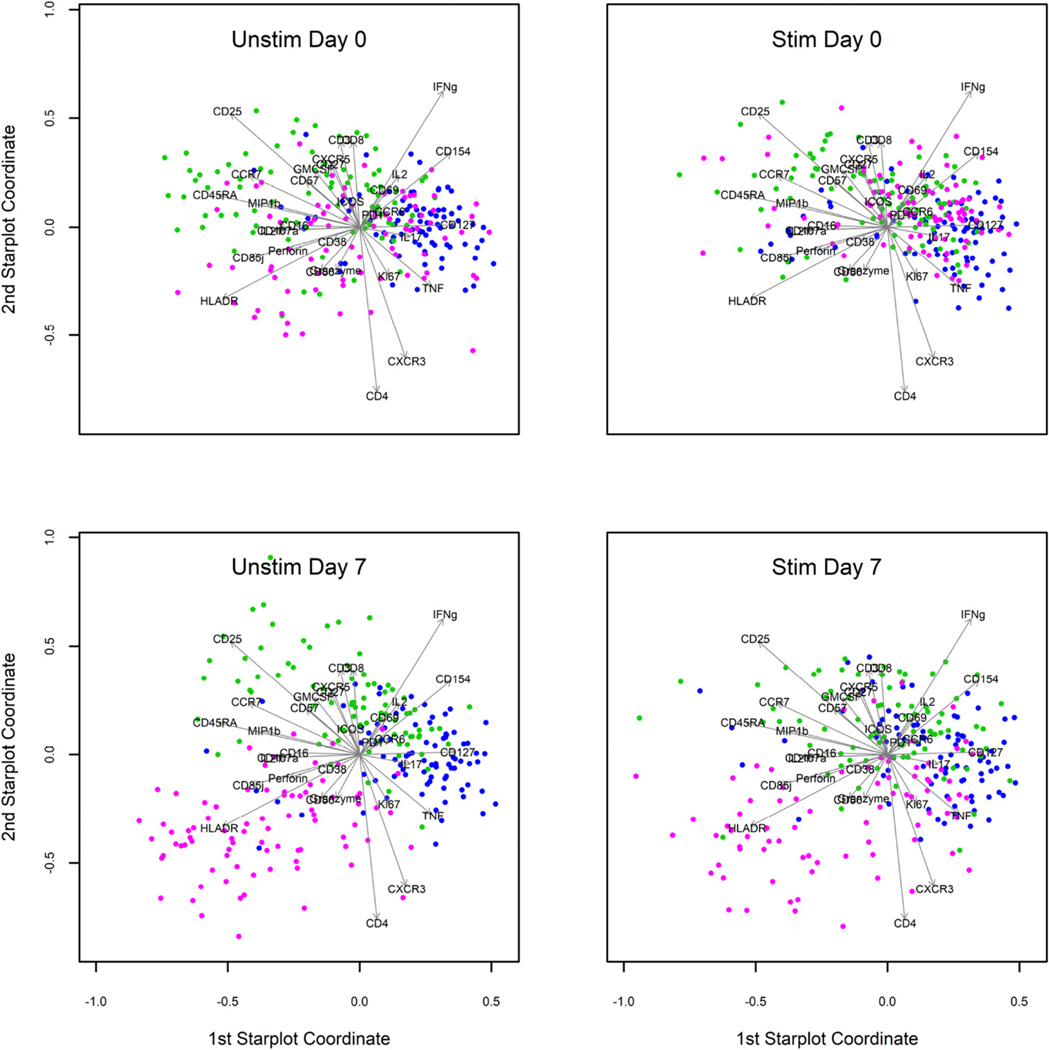
Star plots of three of the six total clusters identified by DRP. Star plots of Clusters 3, 4, and 6 show the expression of various markers represented in two-dimensional space. Individual cells from each cluster are represented on the star plot as colored dots (3, blue; 4, green; and 6, magenta). Arrows indicate the expression of individual markers. Clockwise from top left shows day 0 unstimulated, day 0 stimulated, day 7 stimulated, and day 7 unstimulated. Star plots were produced through stratified random downsampling to 82 cells for each combination of cluster, visit, and condition, which allowed equal weighting of all three clusters for both visits and both conditions. For this reason, these star plots can only be used to assess patterns in expression and not in abundance. Software packages for producing star plots were base R plus R packages matrixcalc, plotrix, JPEN, sampling, and VCA, as indicated in the Materials and Methods. Stim day 7 modified from Holmes et al. (34) with permission from Mary Ann Liebert, Inc., New Rochelle, NY. Stim, stimulated with HA1NP peptide mix; Unstim, unstimulated.

Our data showed that Cluster 3 appeared to be influenza virus specific in that it was significantly more abundant with influenza peptide stimulation versus the unstimulated condition at both time points (Fig. 2, Tables III, IV). In contrast, Cluster 4 was vaccine induced, as it was significantly higher in the stimulated condition at day 7 postvaccination compared with day 0. To explore the phenotypic and functional differences between these clusters, we created star plots (Fig. 3). These star plots are mainly used to assess patterns in expression–qualitative differences beyond abundance. From the star plots, it is apparent that Cluster 3 is very different from Cluster 4, mainly in its high expression of cytokines like IFN-γ and TNF-α with CD154 and CD127. Cluster 4, in contrast, projects more along a complex combination of vectors representing the CD25, CD45RA, CCR7, MIP1β, and GM-CSF, although the precise expression patterns warrant additional visualization methods. Another interesting observation was that Cluster 6 appears to change between day 0 and day 7 postvaccination in both the unstimulated as well as stimulated conditions. We see that Cluster 6 is spread out during the day 0 time point but starts to project along the lower left quadrant that represents HLA-DR, perforin, and granzyme B postvaccination. Thus, Cluster 6 has undergone changes in its marker expression profile following influenza vaccination. However, Cluster 6 did not significantly change in abundance between time points and stimulation conditions (Table IV).

**FIGURE 4.**
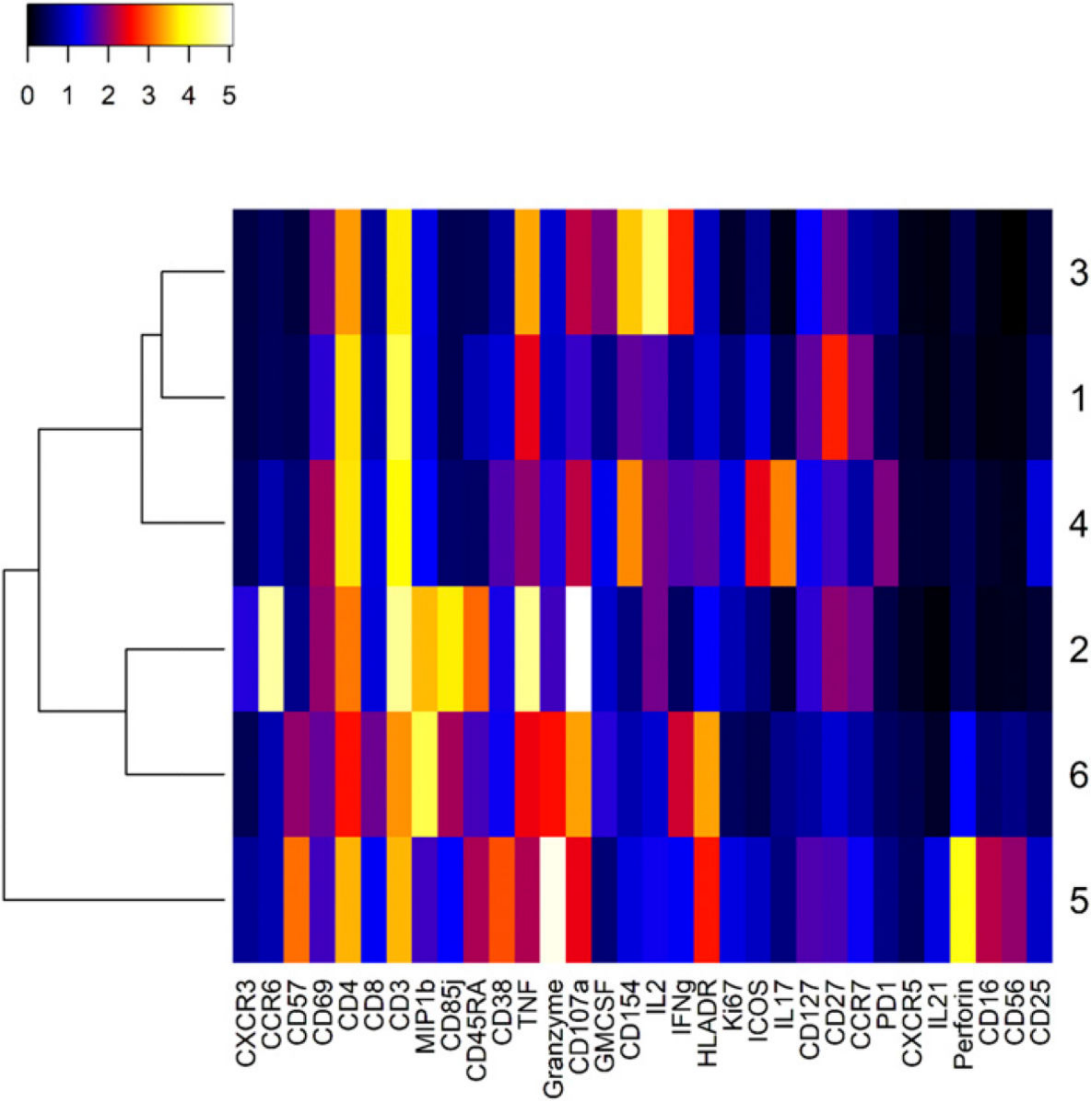
Heat map of expression profiles for all six Clusters. The heat map shows mean expression level [Arsinh (*y*/5), *y* = raw expression] across stimulation conditions and visits in color scale that ranges from white (high) to black (low) for all markers on the panel (labeled at bottom). Cluster label numbers are shown on the right. This heat map allows approximate determination of the mean phenotypic and functional characteristics of the cells that constitute the represented clusters. The heat map was produced with R package heatmap3, as indicated in the Materials and Methods.

**FIGURE 5.**
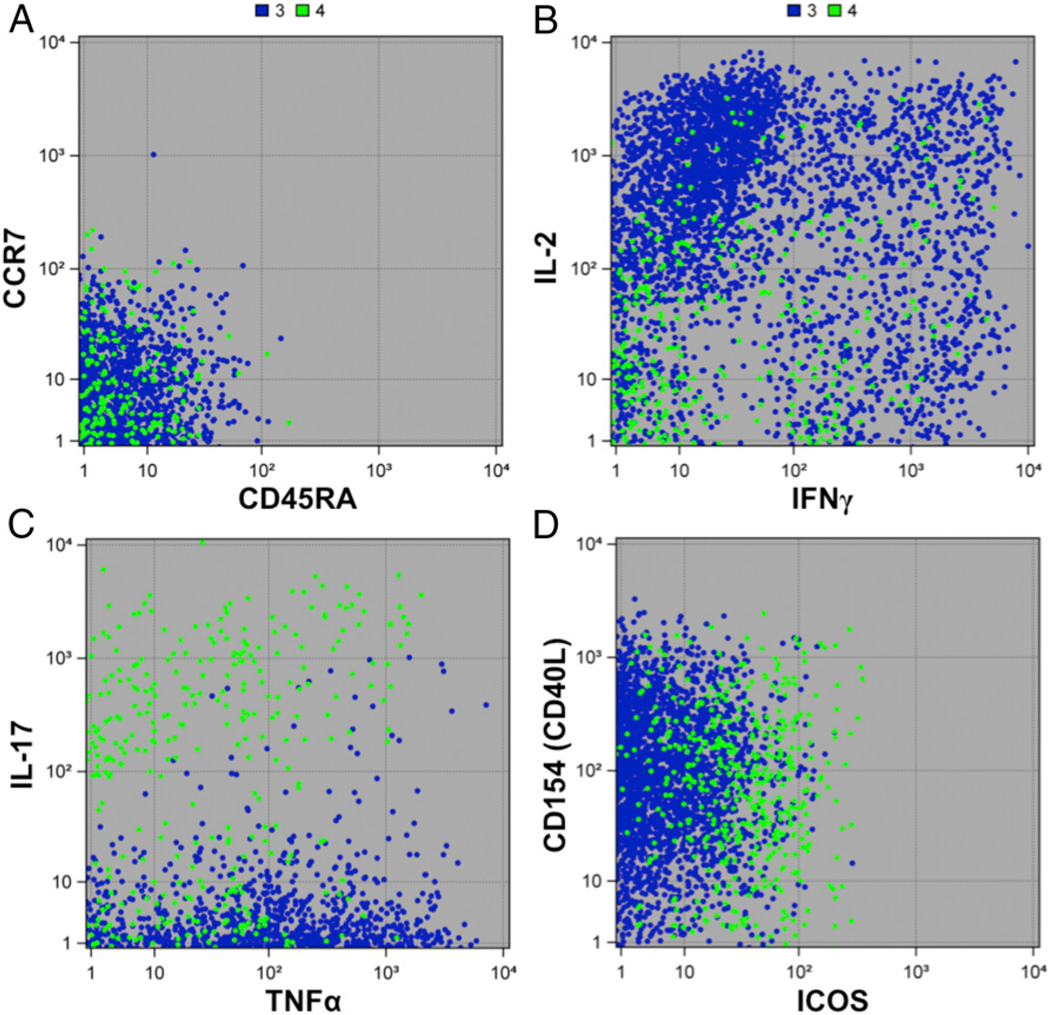
Expression profiles of Cluster 3 and Cluster 4 represented as dot plots. The expression (raw intensity) of some important characterizing markers expressed by Cluster 3 (blue) and Cluster 4 (green) are shown in the form of dot plots. Each dot is an individual cell. Markers shown are (**A**) CCR7 and CD45RA, (**B**) IL-2 and IFN-γ, (**C**) IL-17 and TNF-α, and (**D**) CD154 and ICOS. The figure was prepared in SAS ODS Graphics (SAS Institute).

To further characterize the differences between these influenza virus–specific clusters, we created a heat map of mean marker intensity per cluster across stimulation conditions and visits (Fig. 4). From this heat map, we could discern the major markers distinguishing each cluster. The heat map allows the visualization of all markers at once, but we have also shown the cell-level distribution of expression (pooled across stimulation conditions and visits) for major markers in the form of dot plots (Fig. 5). Both Cluster 3 and 4 showed low expression of CCR7 and CD45RA, indicating that these are most likely effector memory CD4^+^ T cell subsets (Figs. 4, 5A). Both clusters also showed high expression of the activation marker CD154, which indicates an ability to provide help via the CD40L/CD40 pathway (Figs. 4, 5D). Cluster 3 showed high levels of IFN-γ, TNF-α, and IL-2, in addition to the low levels of CCR7 and CD45RA, suggesting that these were functional, cytokine-producing effector memory CD4^+^ T cells (Figs. 4, 5B, 5C). Cluster 4 was also an effector memory-like subset and expressed high CD154 (Figs. 4, 5A, 5D). However, its cytokine profile was very different from Cluster 3, with low levels of IFN-γ, IL-2, and TNF-α and a high level of IL-17 (Figs. 4, 5B, 5C). This indicates that Cluster 4 may be a Th17-like effector memory subset. Cluster 6 showed a high expression of granzyme B and CD107a, in addition to HLA-DR and cytokines IFN-γ, MIP1β, and TNF-α (Fig. 4). Interestingly, among the six total CD4^+^ T cell clusters identified, we observed that some clusters showed cytotoxic markers. Clusters 2, 5, and 6 showed high CD107a levels, Clusters 5 and 6 had high granzyme B, and Cluster 5 had high perforin expression (Fig. 4). This indicates that there are cytotoxic CD4^+^ T cell subsets among the total cytokine-producing CD4^+^ T cells that we analyzed in this study.

### Distribution of clusters within individuals

**FIGURE 6.**
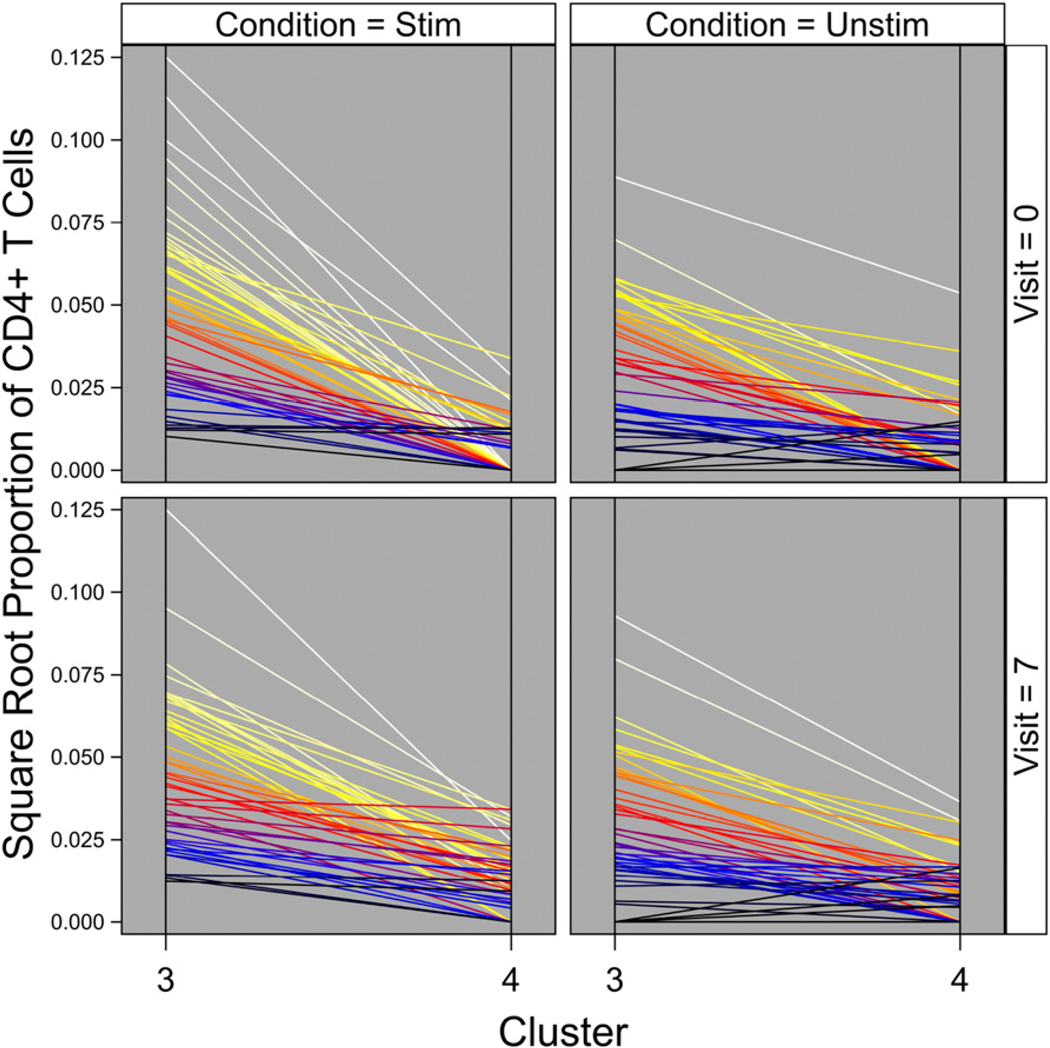
Distribution of Cluster 3 and Cluster 4 within each participant. The distribution of cluster abundance (square root of proportion of CD4^+^ T cells) for Clusters 3 and 4 is shown by participant, separately for each combination of visit and stimulation condition. Each line represents a single study participant. Color-coding is by individual to show how the abundance of cells differs between clusters for that individual. Square-root transformation (vertical axes) facilitates visual separation of individuals. The figure was prepared in SAS ODS Graphics (SAS Institute). Stim, stimulated with HA+NP peptide mix; Unstim, unstimulated.

We next investigated the distribution of Clusters 3 and 4 by study participant to determine if there were any trends in the abundance of the two clusters within an individual (Fig. 6). Some participants had negligible levels of both clusters. Another group of participants had detectable levels of both clusters, but high abundance of Cluster 3 and low abundance of Cluster 4. These distinct subgroups with specific trends in the abundance of Cluster 3 and Cluster 4 reflect an underlying heterogeneity in individual biology. However, abundance of Cluster 3 or Cluster 4 did not correlate with HAI response to the vaccine (data not shown).

## DISCUSSION

With the advent of CyTOF, there has been a surge of single-cell proteomic data on the phenotype and function of immune cells. Having a staining panel of 37 different Abs presents an invaluable opportunity to discover novel cell subsets and understand their biological role. However, analyzing this high-dimensional dataset poses several challenges, which must be overcome by advanced computational methods (54). The DRP method uses a multifaceted approach consisting of denoising the input data, agglomerative hierarchical clustering, and repeated pruning to obtain an optimized cluster solution (Fig. 1).

Rather than denoising, Phenograph addresses the problem of detecting rare phenotypes in noisy data using a two-step procedure for construction of nearest-neighbor graphs (55). The denoising step of DRP relies upon optimal selection of the quantity of principal components to be retained for the hierarchical clustering. Because principal components are linear combinations of the marker intensity values, nonlinear dimension reduction methods, such as *t*-distributed stochastic neighbor embedding (56) and kernel-based density estimation extensions such as automatic classification of cellular expression by non-linear stochastic embedding (57), might identify phenotypes not recovered by DRP. In our experience, *t*-distributed stochastic neighbor embedding did mostly provide clear recovery of subsets in this study’s dataset (Supplemental Fig. 3). Further, we do recommend and did apply a nonlinear transformation of the marker intensity values prior to estimating principal components. Although beyond the scope of the current study, direct comparison of results from DRP and PhenoGraph in several simulated and real small datasets (≤10,000 cells in total) would be highly instructive.

This study did not statistically correct acquired CyTOF data for the nonspecific binding artifact of cross-reactivity. However, our CyTOF panel has been optimally titrated as described (58) to minimize nonspecific binding and spillover.

The DRP method described in this study allowed us to use clustering to identify novel cell subsets and gain a deeper understanding of their phenotypic and functional characteristics. Recall that a “sample estimate” is defined as the value of a parameter estimated from a sample drawn from a population (e.g., sample mean is an estimate of the population mean). The analysis pipeline presented in this study first estimates what clusters of cells are present and, using those estimated clusters, then estimates differences in the mean proportions of each of those clusters between visits and between stimulation conditions. Note that the second set of estimates (differences in mean proportions) thereby depends upon the first set of estimates (cluster identities). Especially rigorous control of type I error (false positives) would propagate estimation (sampling) error in estimates of cluster identities into subsequent estimates of differences in the mean proportions of each of those clusters between visits and between stimulation conditions. How to accomplish this error propagation with out resorting to computationally prohibitive methods requires study and is beyond the scope of the present paper. As such, the comparisons of each cluster’s mean proportions between visits and between conditions that are reported in the present paper should be regarded as liberal (i.e., reject the null hypothesis too often) to some unknown extent. In this study, we focused on CD4^+^ T cells, whose role is not completely understood in the context of influenza vaccination. Using DRP on our dataset, we identified two cell clusters that either responded to influenza peptide stimulation or influenza vaccination (Fig. 2, Table IV). Cluster 3 was significantly above background at both day 0 and day 7, reflecting pre-existing influenza virus–specific cells that presumably persisted from previous vaccination(s) or infection(s) (Table IV). The abundance of Cluster 3 did not change between time points for either stimulation condition. Cluster 4, in contrast, was significantly more abundant with stimulation at day 7 following vaccination compared with stimulation at day 0 (prevaccination) (Fig. 2, Table IV). However, the abundance of Cluster 4 did not increase between the influenza peptide–stimulated condition and the unstimulated condition at either time point. This implies that it is a CD4^+^ T cell cluster that is responsive to influenza vaccination but may not be responsive to the specific peptides used for stimulation in our experiments. Alternatively, the *p* values (adjusted for multiple comparisons) for stimulated versus unstimulated conditions may have not been significant because of the number of cells sampled and/or because of stringent corrections for multiple comparisons (Table IV).

Cluster 3, the pre-existing influenza virus–responsive CD4^+^ T cell subset, showed low CCR7 and CD45RA. This cluster expressed cytokines like IL-2, TNF-α, and IFN-γ, as well as high levels of CD154 (Figs. 4, 5). This corresponds to a functionally ready effector memory subset that is present prior to TIV immunization. The abundance and reactivity of this cluster may depend on factors like previous vaccine experience and the Ag exposure history of the participants.

Both Clusters 3 and 4 seem to be effector memory subsets with low CCR7 and CD45RA expression (Figs. 4, 5A). Their cytokine expression profiles were distinct as Cluster 3 showed high IL-2, TNF-α, and IFN-γ, whereas Cluster 4 mainly expressed IL-17 (Figs. 4, 5). These detailed analyses of Clusters 3 and 4 underscore theroleofCD4^+^memoryTcellsubsetsininfluenzavirusinfection. We also noted that both Cluster 3 and Cluster 4 had high expression of CD154 or CD40L (Fig. 4). This could potentially mean that they can help CD8^+^ T cell activation by dendritic cell licensing through the CD40L/CD40 pathway (59, 60). In addition, we also observed that Cluster 4 showed some expression of ICOS and PD-1 compared with other clusters (Fig. 4). This suggests that these may be Tfh-like cells, although we lacked other Tfh markers or the ability to functionally verify this. We did not see correlations of either of these two clusters with HAI response (data not shown). This could be due to heterogeneity in responsiveness in the sampled population or because the HA peptides used to stimulate CD4^+^Tcell responses represent only a subset of the HA Ags used in HAI assays. Furthermore, the insensitivity/inaccuracy of the HAI assay, interval-censoring of serial dilution data, or the complexity of factors, including participant vaccine history and individual variation, and missing data could influence results for HAI titers. In any case, these influenza virus–specific memory T cells might contribute to protection from disease. This is in accordance with previous studies that have shown that they play a protective role, even in the absence of B cells and CD8^+^ T cells (61).

Clusters1,2,5, and 6 were not influenza virus responsive in that they did not significantly increase in abundance with influenza virus peptide stimulation (Table IV). These clusters also did not increase significantly after influenza vaccination. Most of these clusters were very rare (Table III), and therefore, we may not have collected enough cells to find significant differences from background. It was previously shown that cells producing cytokines in the absence of in vitro stimulation are biased toward a late effector phenotype and are enriched in CMV-reactive cells (in CMV-positive individuals) (62). Cluster 6 was initially of some interest because of an inverse correlation between California strain HAI titer and Cluster 6 abundance at day 0 but not day 7 (Supplemental Fig. 4). However, this correlation was possibly driven by a single extreme value, which made it hard to evaluate the validity of this finding. Additionally, there was some evidence that Cluster 6 may be responsive to the influenza vaccine, based on visual inspection of the star plots (Fig. 3). However, the abundance of Cluster 6 did not change significantly before and after vaccination (Fig. 2, Table IV). Cluster 6 also did not increase in abundance following influenza virus peptide stimulation (Table IV). Heat maps from expression data pre- and postvaccination did not show major changes (data not shown), indicating that the star plots were possibly picking up subtle and complex shifts in its marker expression profile (34). From the heat map, we saw that Cluster 6 had a late effector phenotype with expression of cytokines like MIP1β, TNF-α, and IFN-γ (Fig. 4). This cluster also expressed CD107a and granzyme B. It is possible that our experimental limitations precluded the identification of changes in the abundance of Cluster 6 between stimulation conditions and time points. Alternatively, the change in the expression profile of this cluster (Fig. 3) might be related to changes taking place in other influenza-specific subsets. Without knowing if Cluster 6 is influenza virus–specific or vaccine responsive, it is difficult to fully understand the implications of its qualitative transformation following influenza vaccination. However, given the marker expression profile, it is interesting to speculate that this could reflect a relationship between late effector CD4^+^T cell accumulation in CMV-positive individuals and poor response to influenza vaccine, as previously described (63).

In this study, statistical testing for differences in marker expression levels was not performed because cluster analyses are designed to segregate cells into clusters that are as distinctive as possible with respect to expression. The risk of false positives can therefore become inflated if testing for differences in expression is performed in the same sample used for clustering. As such, a reliable test of differences in expression levels among clusters would need to be performed in a new independent sample of cells.

It would also have been interesting to characterize CD8^+^ T cell responses in addition to CD4^+^ T cells. However, we did not conduct any clustering or analysis of influenza virus–specificCD8^+^ T cells because of low responding cell numbers. In this study, we used a limited set of influenza peptides for stimulation, and it is possible that some responses were missed. This may also explain the lack of expansion of CD4^+^ T cells. Overall, the observable responses are limited to the set of influenza peptides that we used for stimulation.

Another interesting finding from this study was that different participants seemed to have different distributions of the influenza virus–responsive clusters (Fig. 6). Some participants had a low abundance of both clusters, suggesting they were simply poor responders. Of those with higher responses, there tended to be a pattern, with a higher abundance of Cluster 3 and a lower abundance of Cluster 4. Such variations in influenza virus–specific clusters could be due to exposure history or other unknown host factors. Such host differences could affect differential levels of protective responses to influenza vaccination, as seen especially in the elderly.

## Supplementary Material

1

## Figures and Tables

**TABLE I.** Age and gender distribution of participants in study

	Male	Female	Total
Age (y)			
<30	8	7	15
30–39	3	3	6
40–49	3	9	12
50–59	4	7	11
60–69	0	1	1
>70	0	1	1
Total	18	28	46
% of total	39.13%	60.87%	100.00%

Median age of the cohort was 42 y.

**TABLE II.** CyTOF intracellular cytokine staining panel

Specificity	Metal Tag	Source
*Dead cells*	115In	In-house
*Beads*	140Ce	Fluidigm
CXCR3	139La	In-house
CCR6	141Pr	Fluidigm
CD19	142Nd	Fluidigm
CD57	143Nd	In-house
CD69	144Nd	In-house
CD4	145Nd	Fluidigm
CD8	146Nd	In-house
CD3	147Sm	In-house
MIP1b	148Nd	In-house
CD85j	149Sm	In-house
CD45RA	150Nd	In-house
CD38	151Eu	In-house
TNF	152Sm	Fluidigm
Granzyme	153Eu	In-house
CD107a	154Sm	In-house
GMCSF	155Gd	In-house
CD154	156Gd	In-house
IL-2	157Gd	In-house
IFNg	158Gd	In-house
HLA-DR	159Tb	In-house
CD14	160Gd	Fluidigm
Ki67	161Dy	In-house
FOXP3	162Dy	In-house
ICOS	163Dy	In-house
IL-17	164Dy	Fluidigm
CD127	165Ho	In-house
IL-10	166Er	Fluidigm
CD27	167Er	In-house
CD33	168Er	Fluidigm
CCR7	169Tm	In-house
PD1	170Er	In-house
CXCR5	171Yb	In-house
IL-21	172Yb	Fluidigm
Perforin	173Yb	In-house
CD16	174Yb	In-house
CD56	175Lu	In-house
CD25	176Yb	In-house
*DNA1*	191Ir	Fluidigm
*DNA2*	193Ir	Fluidigm

Nonprotein subjects are shown in italics. ^115^In-Maleimide-DOTA, a live-dead stain, and ^191^Ir and ^193^Ir (DNA intercalators) are used to detect live intact singlets. The calibration beads allow us to normalize data for instrument performance.

**TABLE III.** Complete list of estimated proportions of all clusters across both time points and both stimulation conditions

Cluster	Days Since Vaccination	Stimulation Condition	Estimate	SE	Lower 95% Confidence Bound	Upper 95% Confidence Bound
1	0	Stim	0.005287	0.000854	0.003834995	0.007285690
1	0	Unstim	0.004563	0.000921	0.003053719	0.006812640
1	7	Stim	0.005154	0.000842	0.003723868	0.007128893
1	7	Unstim	0.004473	0.000900	0.002998645	0.006668867
2	0	Stim	0.000204	0.000090	0.000084928	0.000489135
2	0	Unstim	0.000148	0.000065	0.000061416	0.000355026
2	7	Stim	0.000199	0.000084	0.000085881	0.000460072
2	7	Unstim	0.000152	0.000068	0.000062763	0.000368067
3	0	Stim	0.003910	0.001195	0.002128751	0.007171356
3	0	Unstim	0.001666	0.000547	0.000867630	0.003197555
3	7	Stim	0.003798	0.001178	0.002049616	0.007026867
3	7	Unstim	0.001645	0.000550	0.000846333	0.003194069
4	0	Stim	0.000253	0.000065	0.000151905	0.000422264
4	0	Unstim	0.000442	0.000131	0.000245043	0.000798285
4	7	Stim	0.000292	0.000069	0.000181568	0.000468155
4	7	Unstim	0.000439	0.000125	0.000249725	0.000772443
5	0	Stim	0.000029	0.000023	0.000006284	0.000137423
5	0	Unstim	0.000018	0.000015	0.000003307	0.000093301
5	7	Stim	0.000029	0.000022	0.000006295	0.000130341
5	7	Unstim	0.000017	0.000014	0.000003317	0.000087339
6	0	Stim	0.000099	0.000046	0.000039411	0.000248397
6	0	Unstim	0.000085	0.000045	0.000029555	0.000243408
6	7	Stim	0.000093	0.000042	0.000037480	0.000228578
6	7	Unstim	0.000075	0.000037	0.000028068	0.000201457

Estimated mean proportions of total CD4+ T cells (and their SEs and 95% confidence intervals) by cluster, days elapsed following vaccination (day 0 or day 7), and stimulation condition. Estimates were obtained from fit of regression model (see Statistical Methods); in contrast to Fig. 2, estimates have been adjusted for covariates. Stim, stimulated with HA+NP peptide mix; Unstim, unstimulated.

**TABLE IV.** All comparisons of mean cluster abundance between time points and between stimulation conditions

Cluster	Comparison of Proportions	Unadjusted *p* Value	FWE Adjusted *p* Value
1	Day 0 Stim minus Unstim	0.0640	1.0000
1	Day 7 Stim minus Unstim	0.0516	0.9292
1	Stim day 7 minus day 0	0.0047	0.0987
1	Unstim day 7 minus day 0	0.0915	1.0000
2	Day 0 Stim minus Unstim	0.4473	1.0000
2	Day 7 Stim minus Unstim	0.4403	1.0000
2	Stim day 7 minus day 0	0.4842	1.0000
2	Unstim day 7 minus day 0	0.7225	1.0000
3	Day 0 Stim minus Unstim	<0.0001	<0.0001
3	Day 7 Stim minus Unstim	<0.0001	<0.0001
3	Stim day 7 minus day 0	0.0306	0.5811
3	Unstim day 7 minus day 0	0.3643	1.0000
4	Day 0 Stim minus Unstim	0.0193	0.3865
4	Day 7 Stim minus Unstim	0.0624	1.0000
4	Stim day 7 minus day 0	0.0009	0.0203
4	Unstim day 7 minus day 0	0.8316	1.0000
5	Day 0 Stim minus Unstim	0.1595	1.0000
5	Day 7 Stim minus Unstim	0.1043	1.0000
5	Stim day 7 minus day 0	0.6446	1.0000
5	Unstim day 7 minus day 0	0.3587	1.0000
6	Day 0 Stim minus Unstim	0.4075	1.0000
6	Day 7 Stim minus Unstim	0.2231	1.0000
6	Stim day 7 minus day 0	0.3127	1.0000
6	Unstim day 7 minus day 0	0.1544	1.0000

For each cluster, comparisons are of mean proportions of total CD4+ T cells between stimulation conditions within each day and, separately, between days within each stimulation condition. Days are days elapsed since vaccination, and *p* values are reported without (unadjusted *p* value) and with (family-wise error adjusted *p* value) correction for multiple comparisons. Comparisons were made from fit of regression model (see Statistical Methods).

FWE, family-wise error; Stim, stimulated with HA+NP peptide mix; Unstim, unstimulated.

## Abbreviations used in this article:

BIIR: Baylor Institute for Immunology Research
CyTOF: mass cytometry by time of flight
DRP: denoised ragged pruning
HA: hemagglutinin
HAI: hemagglutination inhibition
SU: Stanford University
Tfh: T follicular helper
TIV: trivalent inactivated influenza vaccine
