# Supplementary material for: Mass Cytometry Defines Virus-Specific CD4+ T Cells in Influenza Vaccination
Source: Immunohorizons. Author manuscript; Available in PMC 2021 Jun 11. (PMC7891553; doi:10.4049/immunohorizons.1900097)
Supplement: 1 [file NIHMS1668596-supplement-1.pdf]

Supplementary Figure S1

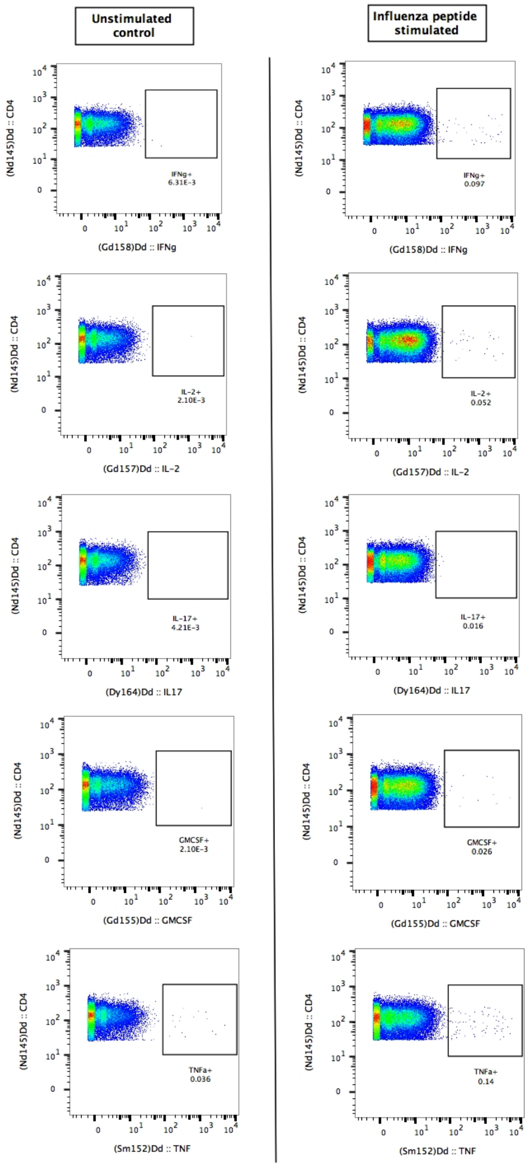

Figure S1. Gating of cytokine producing cells prior to denoised ragged pruning. Total $CD4^+$ T cells were gated for various cytokines. Left column shows unstimulated controls, and right column shows influenza peptide stimulated samples. Top to bottom: IFNγ, IL-2, IL-17, GM-CSF, and TNFα. After gating each cytokine, Boolean logic ($IFN\gamma^+$ OR $IL\text{-}2^+$ OR $IL\text{-}17^+$ OR $GM\text{-}CSF^+$ OR $TNF\alpha^+$) was used to identify any $cytokine^+$ $CD4^+$ T cells. Gating shown from one representative study participant.

Supplementary Figure S2

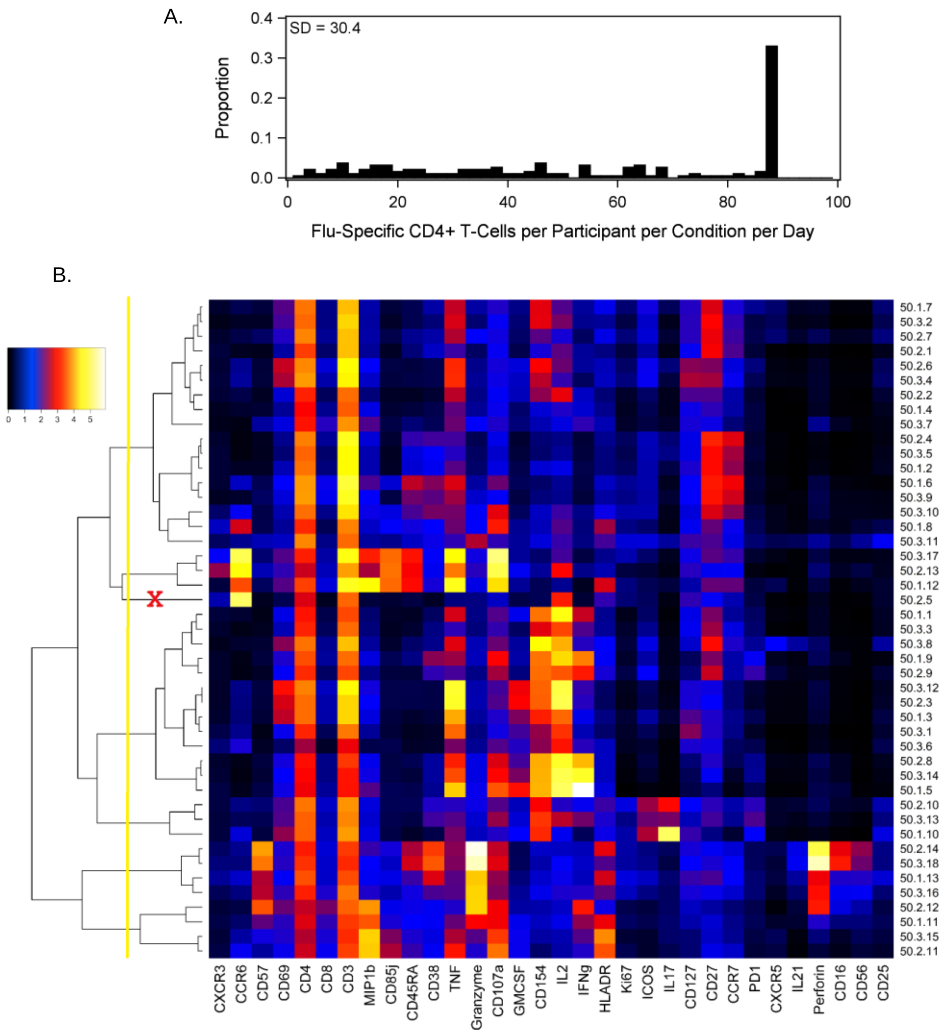

Figure S2. Sampling distribution and final cluster solution after down-sampling. (A) Sampling distribution cell counts per combination of participant, visit, and stimulation condition following stratified random down-sampling. Proportions are of $CD4^+$ T cells. Since participants differ among studies and batches, counts are, more exhaustively, per each combination of study, batch, participant, visit, and stimulation condition. Three separate stratified random down-samplings were performed: results shown are for the third of these three. The spike in the histogram is the result of placing the maximum down-sampling quantity per stratum at 88 cells. Down-sampling was designed to 1) reduce computational memory requirements and time by reducing total quantity of cells input to DRP and 2) reduce inequality in cell counts among strata so that strata contribute less unequally to the DRP cluster solution. All down-sampling was at ~ 50% of original sample size. SD = standard deviation. Figure was generated in SAS® ODS Graphics 9.4. (B) DRP final cluster solution derived from three stratified random 50% down-samplings. Heatmap was produced with R package heatmap3 and edited with Windows® Paint.

Supplementary Figure S3

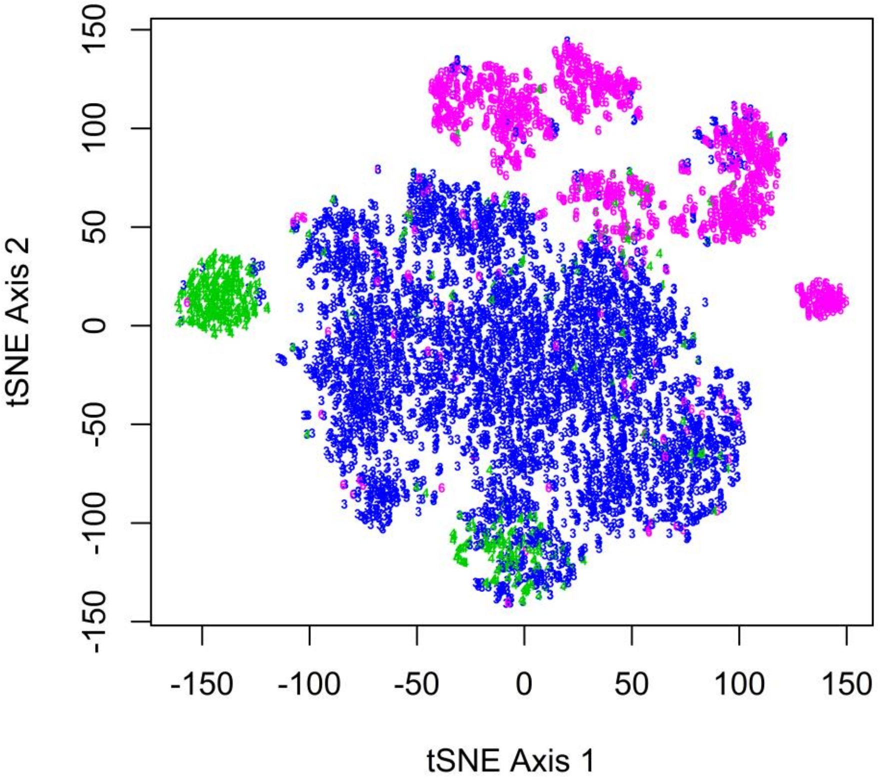

Figure S3. t-SNE plot for final cluster solution. Each datum is a single cell, labeled by cluster assignment: 3 = blue, 4 = green, 6 = magenta. t-SNE partially recovers clusters identified via DRP. Figure was generated using base R (www.r-project.org) and R package tsne.

Supplementary Figure S4

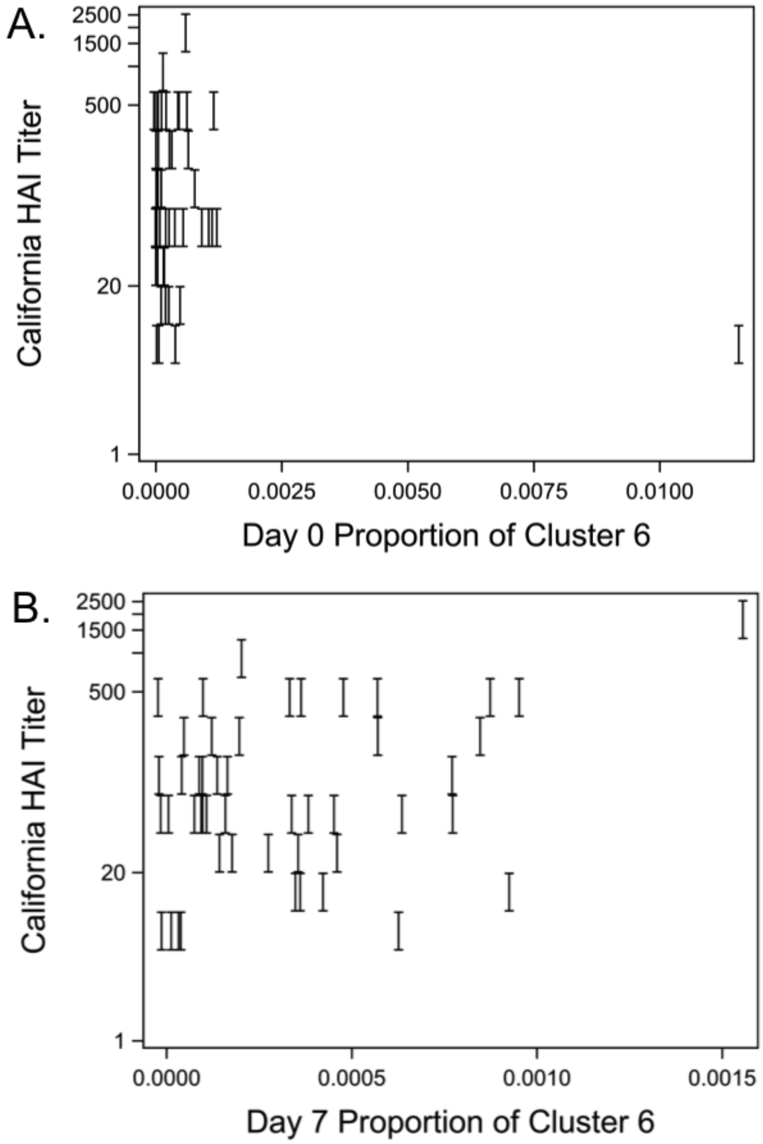

Figure S4. Regression analysis of cluster abundance and HAI titer. HAI titer (California strain) plotted against the proportion of cells of Cluster 6 under the influenza peptide stimulation condition at (A) Day 0 and (B) Day 7. Each I-bar (I) denotes the censored interval of a titer observation in the two-fold dilution series. Figure was generated in SAS® ODS Graphics 9.4 (SAS® Institute, Cary, North Carolina, USA).
